# Mechanisms of inflammation modulation by different immune cells in hypertensive nephropathy

**DOI:** 10.3389/fimmu.2024.1333170

**Published:** 2024-03-13

**Authors:** Xiao-min Hao, Yu Liu, Dilizhawaer Hailaiti, Yu Gong, Xu-dong Zhang, Bing-nan Yue, Ji-peng Liu, Xiao-li Wu, Ke-zhen Yang, Jun Wang, Qing-guo Liu

**Affiliations:** ^1^ Dongzhimen Hospital, Beijing University of Chinese Medicine, Beijing, China; ^2^ School of Basic Medicine, Xinjiang Second Medical College, Karamay, China; ^3^ School of Acupuncture-Moxibustion and Tuina, Beijing University of Chinese Medicine, Beijing, China; ^4^ Department of Chinese Medicine, Beijing Jishuitan Hospital, Beijing, China; ^5^ Department of Rehabilitation Medicine, Sir Run Shaw Hospital, Zhejiang University School of Medicine, Hangzhou, China

**Keywords:** hypertensive nephropathy, hypertension, immunity, inflammation, cytokines

## Abstract

Hypertensive nephropathy (HTN) is the second leading cause of end-stage renal disease (ESRD) and a chronic inflammatory disease. Persistent hypertension leads to lesions of intrarenal arterioles and arterioles, luminal stenosis, secondary ischemic renal parenchymal damage, and glomerulosclerosis, tubular atrophy, and interstitial fibrosis. Studying the pathogenesis of hypertensive nephropathy is a prerequisite for diagnosis and treatment. The main cause of HTN is poor long-term blood pressure control, but kidney damage is often accompanied by the occurrence of immune inflammation. Some studies have found that the activation of innate immunity, inflammation and acquired immunity is closely related to the pathogenesis of HTN, which can cause damage and dysfunction of target organs. There are more articles on the mechanism of diabetic nephropathy, while there are fewer studies related to immunity in hypertensive nephropathy. This article reviews the mechanisms by which several different immune cells and inflammatory cytokines regulate blood pressure and renal damage in HTN. It mainly focuses on immune cells, cytokines, and chemokines and inhibitors. However, further comprehensive and large-scale studies are needed to determine the role of these markers and provide effective protocols for clinical intervention and treatment.

## 1 Introduction

Hypertension is a major public health concern (1), affecting approximately 30% of adults. It is a major contributor to cardiovascular disease morbidity and mortality (2) and is strongly associated with chronic kidney disease(CKD) (3–7). The global prevalence of hypertension-induced chronic CKD exceeds 23.6 million,is characterized by a high degree of hypertension, and severely affects the patient’s quality of life (8, 9). As hypertension progresses, it eventually leads to hypertensive nephropathy(HTN) with irreversible glomerular injury, glomerulosclerosis, tubular atrophy, and interstitial fibrosis (10, 11), which is the second leading cause of end-stage renal disease (12). According to previous studies, the treatment of HTN is relatively limited to controlling blood pressure and regulating lifestyle habits (13, 14). Once the patient develops end-stage renal disease(ESRD), blood pressure control program should be implemented. Notably, dialysis and kidney transplantation are the only available approaches (15), and the treatment of HTN and ESRD is limited. In addition, the incidence of HTN and ESRD is expected to increase in the coming decades (16), placing a heavy burden on healthcare resources.

**Figure 1:**
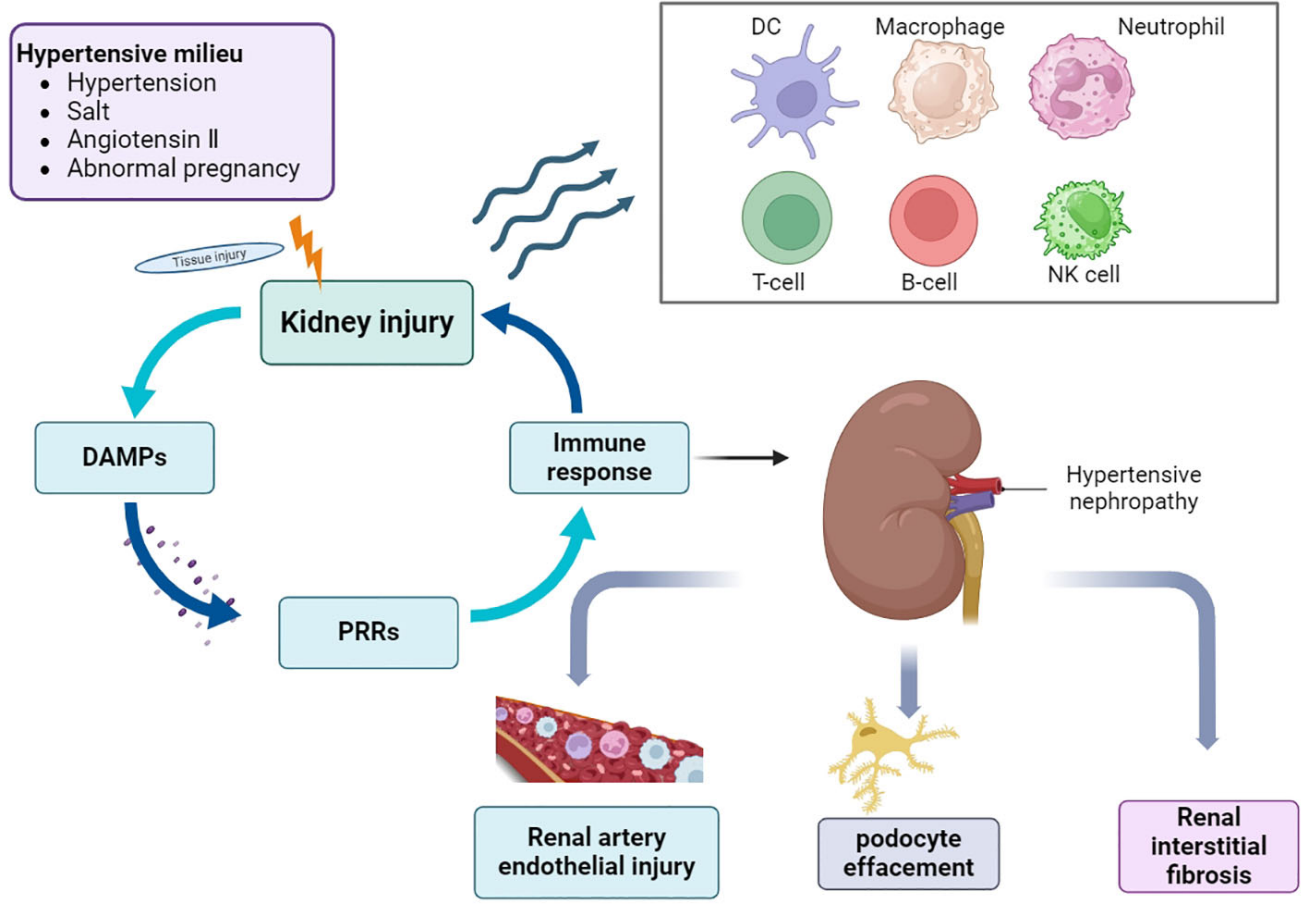
Overview of the pathogenesis of HTN. In the environment of hypertension, hypertension, high salt, angiotensin II and abnormal pregnancy activate a variety of signaling cascades, which promote the recruitment and activation of immune cells, cause the occurrence and development of inflammation, and eventually lead to a series of pathological changes of HTN. DAMPs, damage-associated molecular patterns; PRRs, pattern recognition receptors; DC, dendritic cells; NK cells, natural killer cells.

HTN is first diagnosed based on hypertension and CKD; however, secondary hypertension must be excluded. Hypertension affects the renal vasculature, glomeruli, and tubular interstitium. In general, renal immune cell aggregation promotes an immune-inflammatory response, which in turn disrupts renal blood pressure regulation. Although a large number of studies have been published on HTN in the past, the pathogenesis of hypertension and HTN remains unclear, despite the prevalence of hypertension and associated CKD. Hypertension is closely associated with a continuously activated immune system, which can cause target organ damage and dysfunction, eventually leading to the development of complications such as HTN (17) (**Figure 1**). The first step in innate immune activation that causes hypertension is the pathogen recognition receptor (PRR), which senses a pathogen-associated molecular pattern (PAMP) or damage-associated molecular pattern (DAMP) from stressed or injured tissues and is thus activated to initiate host defense and inflammation (18). Determining the mechanisms by which different immune cells and their subtypes regulate inflammation could help identify new strategies for treating this disease and preventing its progression.

## 2 Relationship between hypertension and kidney disease

Renal injury is a common complication of essential hypertension and is most commonly observed 5–10 years after the onset of hypertension (19). It is also a major cause of kidney disease deterioration. Blood pressure affects the course of kidney disease. A study evaluating ambulatory blood pressure in African Americans found that occult hypertension may be associated with the development of chronic kidney disease (20). Further, insidious hypertension may be associated with the onset of CKD (20). This suggests that regulating blood pressure reduces the risk of hypertension and is also a key factor in reducing the cardiovascular burden of kidney disease (21).

The pathogenesis of CKD is complex, encompassing vascular, glomerular, and tubulointerstitial injuries, and is influenced by environmental and genetic factors, including the severity and duration of hypertension. Remodeling small input arteries is a hallmark of HTN in humans.Hypertension leads to injury and subsequent apoptosis of podocytes, which form the final filtration barrier of the glomerulus, ultimately leading to glomerulosclerosis (22–25).

### 2.1 Small vessel injury

The progression of hypertension leads to changes in small arteries, resulting in intraglomerular hemodynamics changes. A clinical study found that hypertension combined with renal small artery hyalinosis was closely associated with proteinuria and that the accumulation of hyalinized material in the small renal arteries was more likely to lead to hypertensive kidney damage (26). Renal sympathetic nerve activity is usually increased in pathophysiological conditions such as hypertension and chronic and ESRD. Numerous studies (27) have confirmed that elevated sympathetic nerve levels in patients with hypertension lead to deterioration of renal function and abnormal cardiovascular homeostasis due to activation of the renin–angiotensin system (RAS) and renal vascular injury (28, 29). Angiotensin II (Ang II) is most closely related to the RAS (30). Once hypertension occurs, the RAS becomes hyperactivated *in vivo*, potentially leading to glomerulosclerosis and interstitial fibrosis (31). Overproduction of Ang II in the kidney directly constricts vascular endothelial cells (ECs), causing changes in diastolic and contractile substances that are involved in hypertension and kidney injury. In hypertensive rats, the miRNA-mediated mTOR signaling pathway may play a role in Ang II-induced renal artery endothelial cell injury by driving glycolysis and activating autophagy (32).

### 2.2 Glomerular injury

Glomerular injury is a prominent feature of hypertensive nephropathy (33). Any change primarily involving small blood vessels eventually translates into glomerular injury (24). In some clinical studies, patients with a history of hypertension showed a significant reduction in glomerular number (34) and an increase in glomerular volume compared with patients with normal blood pressure (35). Podocytes are epithelial cells that form a glomerular barrier to prevent protein loss (36–39). Podocyte injury is involved in RAS activation and is an essential factor in the progression of HTN and diabetic nephropathy (40). Ang II is a central active molecule of the RAS, plays a role in regulating hypertension-induced inflammation in organ damage (41, 42), and can directly contribute to podocyte injury (43, 44). Podocyte injury was positively correlated with mean arterial pressure, suggesting that higher blood pressure was more likely to cause glomerular injury (45). Hypertensive disease causes podocyte damage and apoptosis, leading to glomerulosclerosis (46). As a pro-apoptotic protein, septin4 is an important marker of organ damage. Recent studies have shown that septin4 promotes hypertensive kidney injury by activating the acetylation of K174 (K174Q). Septin4–K174R is one of the renoprotective factors that ameliorates Ang II-induced hypertensive kidney injury (47). In addition, an increase in blood pressure leads to arterial stiffness, which eventually affects the arteries and causes glomerular lesions (48).

Vascular endothelial growth factor (VEGF), produced by podocytes, is a significant regulator of vascular biology and plays a key role in glomerular development and function (49). Further, it has been reported in various types of glomerulonephritis, including diabetic nephropathy, which is usually associated with increased VEGF-A (50).

### 2.3 Renal tubulointerstitial injury

Sustained hypertension causes renal tubular cell damage and tubulointerstitial fibrosis (TIF). In patients with hypertension, numerous scattered inflammatory cells may trigger TIF (51). Renal interstitial injury is a dynamic and heterogeneous structure involving a variety of molecules, such as collagen, glycosaminoglycans, and glycoproteins. Notably, type I, III, V, VI, VII, and XV collagen are all expressed in the kidney under physiological conditions. The expression of type III procollagen and type V collagen is significantly increased in spontaneously hypertensive rats(SHR) kidneys, suggesting that these collagens play a role in hypertension-induced renal fibrosis (52). Further, complement C3 expression was significantly increased in the renal mesenchymal tissues of SHR, inducing the synthesis and overgrowth of mesenchymal cells (53). Similar results were confirmed in a clinical study in which enhanced interstitial complement C3 expression in patients with HTN was significantly associated with interstitial macrophage density, serum creatinine levels, interstitial fibrosis, glomerulosclerosis, and arteriopathy scores (54). The results of another clinical study revealed (55) that mast cells were mainly present in the renal tubulointerstitium and increased in number in human HTN, suggesting the involvement of renal mast cells in the progression of HTN.

## 3 Role of immune cells in HTN

The complex role of immune system in hypertension was proposed as early as the 1960s (56). Hypertension is a chronic inflammatory disease, and hypertensive stimuli lead to the accumulation of pro-inflammatory cells, such as macrophages and T lymphocytes, which affect the development and progression of hypertension (57). In renal diseases, inflammation, immune cell aggregation, and cell death form a vicious cycle of fibrous matrix deposition that destroys renal tissue structure and ultimately leads to renal dysfunction (58). Immune cells can maintain renal homeostasis and attenuate renal damage. In addition, immunosuppressive agents have been used to treat kidney disease (59). The cells of the innate immune system in renal immunity include dendritic cells (DCs), macrophages/monocytes, natural killer (NK) cells, T cells, B cells, and neutrophils. HTN is an inflammatory disease caused by metabolic disorders in nature (60), which can be manifested as an increase in lymphocyte count (61), thereby increasing the risk of HTN.

### 3.1 DCs and macrophages

Considerable overlap has been reported between DCs and macrophages regarding cell origin morphology, function, and cell surface antigen expression (62). As innate immune cells, DCs and macrophages can act as sentinels and messengers and are closely related to individual development and the triggering of inflammatory responses. Simultaneously, they complement each other in their surveillance functions (63). DCs focus on antigen presentation, whereas macrophages play a role in clearing dead cells and repairing damaged tissues. These cells induce and regulate the inflammatory response and protect the kidney from infection (64).

DCs form a dendritic network that spans the barrier surface and entire organs, including the kidney, performing important sentinel and messenger functions (65). DCs are the most potent antigen-presenting cells (APCs) in the body, located in the interstitial space of the renal tubules, with immature DCs located closer to the vasculature (66). CD103+ cells mainly activate cytotoxic T cells (67), whereas CD11b+ cells activate CD4+ T cells and produce inflammatory chemokines (68). Notably, DCs are the effectors of renal inflammation, as depletion of CD11c+ cells ameliorates antibody-induced inflammation in the glomerular basement membrane (69). Renal DCs mediate the transfer of T cells to the kidney in an antigen-dependent manner during bacterial infection, suggesting that renal DCs not only monitor systemic infections but are also crucial in localized infections and autoimmunity (70). Similar findings were observed in Ang II-induced hypertension. Renal mid-renal DCs also present antigens to T cells under appropriate instructions (71). In hypertensive mice (72), activated DCs improved sensitivity to hypertension; however, only responsive T lymphocytes can trigger activation. In another mouse experiment (73), mice lacking CD70 did not aggregate T (EM) T-cells and did not develop hypertension to the point of attenuated kidney injury under high-salt conditions or a second Ang II challenge. In addition, typical FLT3L-dependent DCs promote T-cell activation in the kidney, leading to oxidative stress, fluid retention, and elevated blood pressure (74).

Macrophages participate in the innate immune response, immune surveillance, and the maintenance of renal homeostasis (75, 76). Macrophages have various functions in different microenvironments (77), responding differently depending on the nature and duration of renal injury (78–80). Macrophage phenotypes are influenced by the microenvironment, ranging from pro-inflammatory (termed classical M1-type activation) to anti-inflammatory or pro-catabolic (termed selective M2-type activation) (81, 82). The main functions are maintenance of homeostasis and phagocytosis to remove various foreign bodies (83, 84). M1 macrophages favor the clearance of infections and promote immune responses (85). M2 macrophages have a wide range of functions that inhibit inflammation and promote tissue repair (86). In the kidneys, immune activation significantly increases the number of macrophages, leading to the infiltration of monocyte-derived macrophages involved in inflammation in the stenotic kidney and the promotion of fibrosis, especially during the chronic phase of kidney disease (87–89). As previously mentioned (54), inflammation with complement C3 activation and macrophage infiltration and their interaction may play an important role in the pathogenesis of human HTN. There are also data confirming (90) that increased macrophage infiltration is closely correlated with increased YM1/Chi3l3 expression, suggesting that YM1/Chi3l3 may be a biomarker for HTN. Recent evidence shows (91) that VEGFC inhibits inflammasome activation by promoting high salt-induced autophagy in macrophages to ameliorate hypertensive kidney injury.

### 3.2 Neutrophils

Neutrophils are the first line of defense in the innate immune system and help maintain blood vessel tone (92). A study found that neutrophils in the aorta increased after 7 days of Ang II-induced hypertension (93). An increase in neutrophil count can aggravate the risk of chronic renal failure. However, direct evidence to confirm the role of neutrophils in HTN remains lacking.

### 3.3 T-lymphocytes

T-lymphocytes are immune cells with the greatest phenotypic and functional diversity. Growing evidence suggests that T cells are involved in the development and progression of HTN. Further, the FASL/FAS (CD95L-CD95) pathway is activated in the kidneys of hypertensive rats, and cytotoxic T lymphocytes (CTLs) kill target cells through this apoptotic pathway. Activation of the FAS/FASL pathway may also be a mechanism underlying renal tubular epithelial cell loss and mesangial fibrosis, which are prominent renal characteristics of hypertensive kidneys (94). Earlier studies (95) also found lymphocytic infiltration in the renal interstitial space in patients with hypertensive renal injury. Some studies have reported (96) that lymphocytes can reduce sodium excretion during Ang II-dependent hypertension by regulating NOS3 and COX-2 expression in the kidneys. Notably, the main source of interferon (IFN)-γ and TNF are CTLs, and mice lacking any of these cytokines showed attenuated hypertension and renal injury in the presence of hypertensive stimuli (97, 98), which aligns with the pathogenic role of CTLs. CTLs play a critical role in hypertension. However, only the CD8+ T-cell population is antigenically activated in the kidneys of hypertensive mice, which may be the main site of immune activation in hypertension (99). This study also found that Cd8 mice had an approximately 50% reduction in the hypertensive response to Ang II or deoxycorticosterone acetate (DOCA) and salt treatment. CD8+ mice had fewer kidney leukocytes than wild-type mice, thinner small renal arteries, and less endothelial dysfunction (99). In a recent study, during DOCA- and salt-induced hypertension, CD8+ T cells acted directly on renal distal tubules to maintain renal retention (100). Therefore, CTLs may influence the blood pressure by enhancing renal reabsorption of sodium and water.

Activation of the immune system contributes to the pathogenesis of hypertension and the progression of chronic kidney disease. An imbalance in the helper T cell subpopulation is associated with Ang II-induced hypertensive kidney injury (101). T regulatory (Treg) cells are key regulators that mediate immunity and are involved in the activation and proliferation of auto T cells, which play an essential role in autoimmune diseases, immune homeostasis, graft tolerance, and immune regulation of tumors (102). In addition, Tregs exert an inhibitory effect on inflammation by secreting the cytotoxic factors, perforin, and granzyme.

The discovery of Tregs has led to a better understanding of CKD and HTN pathogenesis. Notably, the number of Treg cells in the serum of patients with CKD is significantly decreased, and the percentage of Th17 cells, serum IL-17 levels, and ratio of Th17/Treg cells are increased with the progression of CKD (103). Further studies found that transferring Tregs from healthy mice to mice treated for kidney injury attenuated the condition (104). Moreover, macrophages work synergistically with Tregs to attenuate kidney injury by enhancing chemokine expression (105). Tregs also reduce kidney injury by inhibiting macrophage activation and reducing pro-inflammatory cytokines (106). Clinical and basic data have shown that the Th17 pathway has specific effects on hypertensive kidney injury, ultimately leading to kidney injury (107–109). The Th17 pathway has been shown to affect hypertension (110) specifically.

### 3.4 NK and NKT cells

A study found that NK cells play an important role in Ang II-induced vascular dysfunction (111). A laboratory study confirmed that the long-term infusion of IL-17 into pregnant rats results in NK cell activation and proliferation (112). In another study (113), CD1d-dependent NKT cell activation was protective against Ang II-induced cardiac remodeling. However, the specific mechanisms underlying hypertension and concomitant renal disease require further investigation.

### 3.5 B cells and antibody-secreting cells

B cells play a role in hypertension by producing antibodies. For example, the B cell/IgG pair is closely associated with Ang II-induced hypertension and vascular remodeling in mice (114). It has been reported (4, 114) that B-cell activation and IgG production are closely associated with the pathogenesis of hypertension and end-organ damage. Targeting B cells may be able to ameliorate renal lipid peroxidative damage in patients with hyperhomocysteinemia (HHcy) and HTN (115).

Inflammation is a natural and necessary response of the body to stimuli. It is responsible for migrating immune system cells to the stimulus target after a series of steps facilitated and coordinated by cytokines, chemokines, and acute-phase proteins (58). Activation of the immune system is involved in the pathogenesis of hypertension and contributes to CKD progression (116). The accumulation of immune cells in the kidneys promotes a chronic inflammatory response that disrupts blood pressure regulation (117). Inflammation is a key factor in the pathogenesis of hypertension and cardiovascular diseases. Some studies have found that inflammation, immune cell infiltration, and alterations in chemokines and cytokines characterize hypertensive kidney damage (118, 119).

## 4 Cytokines and HTN

### 4.1 Definition and function of cytokines

Cytokines are small proteins involved in the development and activity of the immune system, have a wide range of biological activities, and help coordinate the body’s response to infection (120, 121). Hypertension is associated with the aggregation of T cells and monocytes/macrophages in the blood vessels and kidneys, which produce potent cytokines that affect vascular and renal function (122). Innate immune cells express several receptors that recognize microorganisms, induce rapid defense, and delay cellular responses. Toll-like receptors on the cell surface and endosomal membranes monitor extracellular microbes and activate multiple host defense signaling pathways (123). The NF-κB and interferon regulatory factor (IRF) pathways downstream of these receptors induce the expression of pro-inflammatory cytokines TNF-α, IL-1β, and IL-6, among others (**Table 1**).

**Table 1:** Cytokines involved in HTN pathogenesis.

Cytokines	Cell Source	Functions in kidney disease	References
TNF-α	Macrophages, monocytes, T cells	Increase the permeability of vascular endothelial cells	(124, 125)
IL-1	Monocytes, macrophages, fibroblasts epithelial cells	Accelerate renal fibrosis	(126–129)
IL-6	T cells, macrophages, fibroblasts	Accelerate renal immune damage	(130, 131)
IFN	T cells, NK cells	Aggravate glomerular injury	(132, 133)
IL-10	T cells	Improve vascular injury	(134–136)
IL-17	T cells	Reduce kidney inflammation	(137, 138)
TGF-β	Macrophages, T cells	Accelerate renal fibrosis	(139–141)

### 4.2 Role of cytokines inHTN

TNF-α, a typical macrophage factor appearing earliest during the inflammatory response, is another cytokine released by immune cells (142). TNF-α activates neutrophils and lymphocytes, increases the permeability of vascular endothelial cells, regulates tissue metabolism, and promotes cytokine synthesis and release (143, 144). When inflammatory lesions occur in the kidney, mononuclear macrophages are infiltrated and can express and secrete large amounts of TNF-α, promoting the secretion of IL-6 by the tethered cells. Many experimental studies have found that TNF-α contributes to the proliferation of tethered cells and stroma and the widening of tethered zones, which promotes the onset and development of inflammation. For example, TNF deficiency in mice attenuates the Ang II-induced hypertensive response and renal injury (97, 116, 124). TNF is toxic to glomerular epithelial cells (125, 145), regulates blood pressure, and ameliorates the extent of renal injury (146–149). Inflammatory factors such as TNF-α, IL-1β and MCP-1 were significantly expressed in the kidney of SHR (150). In humans, the effect of TNF on blood pressure is more complex. For example, in patients with heart failure, TNF antagonism does not improve blood pressure or symptoms (151, 152).

In contrast, in animal models of CKD, TNF elevates renal and systemic FGF23 levels, which may be involved in CKD (153). Similarly, in an animal model of diabetic nephropathy, it was found that macrophage-produced TNF-α plays a role in diabetic kidney injury, mainly reducing albuminuria, plasma creatinine, histopathological changes, renal macrophage recruitment, and plasma inflammatory cytokine levels (154). In a mouse study (116), TNF in the kidney elevated blood pressure by reducing NO production. In other experiments, TNF-α was downregulated through Cyp2c23 and upregulated through sEH (148), activation of NF-κB-mediated pathways (147), and increased renal cortical NF-kappa B activity (149) that are involved in renal damage caused by multiple causes of hypertension. In rats (146), the direct infusion of TNF inhibitors into the renal interstitium attenuated salt-induced blood pressure elevation and renal damage. Another study confirmed (126) that during prolonged administration of Ang II and hypertension on a high-salt diet, TNF receptor 2 may mediate the inflammatory response to renal injury without activating TNF receptor 1. Therefore, TNF-α is an essential mediator of hypertension and kidney injury.

IL-1 acts as an inflammatory cytokine with two active isoforms, IL-1α and IL-1β, which are recognized by IL-1 receptor. NLRP3 is an inflammatory vesicle that promotes the maturation and secretion of IL-1β and IL-18 (127–129, 155). IL-1β can act on pericytes to accelerate renal fibrosis (156) and activate helper T cell 17 (TH17) cells in the kidneys (157). Activation of NLRP3 inflammatory vesicles causes elevated blood pressure and/or kidney injury (158–161), exacerbating cardiovascular risk (128). The guanidinylation of apolipoprotein C3 (ApoC3) activates NLRP3 inflammatory vesicles in human monocytes (162). Guanidinylated ApoC3 (gApoC3) accumulates in the kidneys and plasma of patients with CKD to promote inflammation. gApoC3 promotes renal fibrosis and impedes vascular regeneration (163). Mechanistically, NLRP3 directly promotes the epithelial-to-mesenchymal transition of renal tubular epithelial cells, independent of inflammatory vesicles, by inducing phosphorylation and activity of SMAD3 (164). In SHR, BCL6 attenuates renal inflammation by negatively regulating NLRP3 transcription (165). In a recent study (91), VEGFC was shown to inhibit the activation of NLRP3 inflammatory vesicles by promoting autophagy to ameliorate sensitized hypertension and nephritis, which may be a potential therapeutic target for HTN. We found that the infusion of exogenous IL-1 promotes urinary sodium excretion (166–168). Further, IL-1 is involved in developing pulmonary or systemic hypertension through several other mechanisms (169, 170). Several studies have also reported the use of IL-1 inhibitors (171). For example, the IL-1 receptor antagonist anakinra attenuates hypertension and renal fibrosis but does not affect inflammation and leukocyte infiltration in saline-induced hypertensive mice (1K/DOCA/salt) (172). Therefore, investigating the renal-specific mechanisms of IL-1 in hypertension is crucial.

IL-6, known for its pro-inflammatory effects, is one of the most studied cytokines in renal disease and plays a role in systemic inflammation (121, 130). IL-6 has been reported to be involved in hypertension-induced kidney injury (131, 173). IL-6 induces B-cell differentiation and antibody production as a pro-inflammatory response, causes T-cell proliferation and differentiation, and regulates the immune response. IL-6 promotes the expression of cell adhesion molecules by inducing cytokine production of acute-phase response proteins, which leads to the aggregation of inflammatory cells. The expression of IL-6 is directly proportional to the number of glomeruli in glomerular disease. In addition, it stimulates the production of platelets by tethered cells activating factors and other inflammatory mediators, exacerbating renal immune damage. Notably, the risk of cardiovascular death, myocardial infarction, and stroke is associated with decreased glomerular filtration rate (eGFR) and elevated IL-6 levels (174). IL-6 levels in CKD are involved in atherosclerosis (175, 176). Notably, the results of these studies are mutually contradictory. Other studies also reported conflicting findings, such as that IL-6 levels at baseline or changes during the observation period do not show a consistent and significant correlation with renal function in older adults (177). Recently, drug studies on IL-6 and IL-6 receptors have also gained momentum (121, 132, 178–180).

IFN is an inflammatory cytokine produced by T lymphocytes and macrophages that enhances sodium transport by activating intrarenal Ang II production to stimulate transport proteins (133) indirectly. Therefore, IFN deficiency attenuates the response to Ang II-induced chronic hypertension (98) and exacerbates glomerular injury (181). IRF is a multifunctional transcription factor, and IRF-4 deficiency improves renal function, albuminuria, and attenuates renal injury and fibrosis but does not affect blood pressure (182). IRF-4 is a multifunctional transcription factor.

Cytokines in the kidneys act indirectly by inducing other inflammatory mediators and their direct effects on tissue cells. NF-κ B is a critical transcription factor in regulating the inflammatory response and the expression of many pro-inflammatory genes (183). The complex hypertensive environment (i.e., hypertension, oxidative stress, glomerular leakage of free fatty acids, and activated RAS) can exacerbate renal inflammation and the progression of HTN by activating the NF-κB signaling system (184, 185). During glomerular disease progression, NF-κB activation triggers an inflammatory cascade that produces inflammatory factors, such as IL-10 and MCP-1.Studies have found (186) that TGF-β/SMAD and NF-κB signaling pathways play an important role in HTN, and SMAD7, as a downstream inhibitor of these two pathways, can inhibit proteinuria and serum creatinine, and improve glomerular filtration rate, thereby alleviating the condition of HTN. In a recent study (187), ubiquitin-specific protease 25 (USP25) inhibited TGF-β-induced SMAD4 ubiquitination, which reduced the expression of renal fibrosis and renal injury related genes and alleviated renal injury caused by HTN. It has also been confirmed (134) that exercise training may prevent and alleviate renal abnormalities in hypertensive patients by down-regulating TGF-β signaling. Renal fibrosis is an important pathological feature of hypertensive renal injury, which is related to the up-regulation of pro-fibrotic factor TGF-β1 (150).

IL-10 is strongly associated with inflammation in the kidney (135, 136, 188). For example, IL-10 ameliorates hypertension-induced renal and vascular injury (189), and genetic polymorphisms in IL-10 are also protective against diabetic nephropathy (139). MCP-1 promotes chemotaxis of monocytes or macrophages to reach damaged renal tissues, resulting in sustained cytokine activation and localized protease disruption of the renal basement membrane. MCP-1 increases the production of IL-8 and TGF-β1 by affecting the inflammatory response of diseased renal tissues. TGF-β, a cytokine that can regulate cellular proliferation, plays a role in the process of membranous nephropathy, diabetic nephropathy, and renal fibrosis (140, 141, 190).

Chemokine ligand 16 (CXCL16) plays a role in Ang II-induced renal injury and fibrosis by regulating macrophage and T cell infiltration and fibroblast aggregation (191). During RAS-activated hypertension, the chemokine CCL5 attenuates renal injury and fibrosis by reducing the number of infiltrating macrophages in the kidneys (137).

IL-17 is also involved in Ang II-induced hypertension and vascular function (138). In a mouse study (192), IL-17A and IL-17RA antibodies (but not IL-17F) reduced blood pressure and attenuated renal and vascular inflammation. Notably, in other experimental models of kidney injury, IL-17 deficiency exacerbated disease progression (193–195). In a recent transcriptomics study (196), genes upregulated in the renal tubules of patients with HTN were associated with IFN-γ, NF-κB, IL-12, and Wnt signaling pathways, all of which are involved in the inflammatory response, providing a broader perspective on the pathogenesis and treatment of human HTN. Although the number of immune cells is small, the released cytokines play key roles in renal changes involved in fibrosis, glomerular injury, and sodium transport (58).

## 5 Oxidative stress and HTN

The signaling pathways between oxidative stress and HTN are complex in the pathophysiology of HTN (197, 198). In the kidney, ROS exists in arterioles, glomerular and tubular cells and podocytes, etc. Vasoactive substances and metabolic factors stimulate the production of cellular inflammation and ROS, inducing NADPH oxidase or mitochondrial production (199). The endoplasmic reticulum (ER) is a key player in the redox pathophysiology of the cardiovascular system that maintains protein homeostasis. ER stress (ERS) is typically characterized by impaired protein and lipid synthesis and disturbed intracellular calcium levels (200–202). Excessive ERS damages glomerular mesangial cells, podocytes, and tubular epithelial cells (203–205). However, ERS activity may impair renal function and worsen renal damage during CKD development. Oxidative stress affects T-cell activation and function (206). Septin4 is a non-histone protein that promotes apoptosis, is regulated by SIRT2, and is a marker of organ damage (207–210). SIRT2 deficiency increases oxidative stress and alters mitochondrial morphology (211). Notably, SIRT2–septin4 axis prevents hypertensive kidney injury by attenuating apoptosis and oxidative stress. SIRT2 attenuated oxidative stress in podocytes through deacetylation of septin4-K174, ameliorating hypertension-induced kidney injury in mice. Therefore, septin4-K174R (mimicking deacetylation via SIRT2) may be a new target for alleviating renal injury in hypertension (47), which may help design precise therapeutic regimens and develop targeted drugs in the future. Some studies have demonstrated that SIRT3 is involved in hypertensive kidney injury by inhibiting the epithelial–mesenchymal transition (EMT) and ameliorating hypertension-induced kidney injury in mice (212, 213). Another study in rats (214) suggested that cagliflozin regulates renal EMT and oxidative stress through the SIRT3 pathway, thereby inhibiting high-salt diet-induced hypertensive renal fibrosis.

## 6 Interaction of immune cells and inflammatory factors in HTN

Modern medical research has found that many glomerular diseases are immune-mediated inflammatory lesions. The involvement of various inflammatory mediators, such as inflammatory cytokines and complement factors based on immune response, leads to glomerular injury and the development of corresponding clinical symptoms (215). The immune system plays an important role in developing hypertension and related endpoints, such as the associated end-organ damage. Sodium provides a strong stimulus for the development of hypertension. High concentrations of extracellular natriuretic ions have been reported to directly activate the inflammatory response in APCs, leading to hypertension, T-cell activation, and ultimately vascular and renal injury (216). APCs respond to hypertension-stimulated inflammatory processes, and DCs, macrophages, and B cells are the classical APCs of the immune system, which coordinate the immune response by activating T cells through antigen-MHT receptor complexes and expressing cytokines, among others (217). Large amounts of sodium-activated DCs produce IL-1β and promote the production of IL-17A and IFN-γ by T cells, which in turn lead to mediated hypertension and end-organ dysfunction, indicating that the mechanistic link between salt, inflammation, and hypertension includes increased oxidative stress and IsoLG production in DCs (218). Macrophages and T cells play important roles in developing HTN-induced kidney disease (219). Pro-inflammatory cytokines, tumor necrosis factor-α, IL-6, and IL-1β have been implicated in the pathogenesis of Ang II-induced target organ damage (220). For example, as mentioned previously, the deletion of CXCL16 affects the expression of pro-inflammatory cytokines in the kidney. TGF-β1, a key pro-fibrotic cytokine, causes renal damage and tubulointerstitial fibrosis. One study showed that CXCL16 deficiency caused a significant reduction in Ang II-induced TGF-β1 gene expression, which may be an alternative mechanism for developing renal fibrosis.

GPR97, a member of the G protein-coupled receptor subfamily, modulates the inflammatory response (221, 222). GPR97 exacerbates AKI by regulating Sema3A. GPR97 was expressed in tubular epithelial cells in mice with AKI (223). Recent animal experiments have also found (224) that GPR97 causes renal injury and tubulointerstitial fibrosis (TIF) in deoxycorticosterone acetate (DOCA)/salt-induced hypertensive mice by regulating TGF-β signaling. Therefore, GPR97 leads to TIF in patients with hypertension, indicating that it may be a novel therapeutic target for delaying or attenuating renal injury in hypertension.

## 7 Strategies for prevention and treatment of HTN

HTN, as a cross-discipline, presents difficulties and challenges in diagnosis and intervention. Patients with HTN should monitor and manage their blood pressure and set appropriate control goals in their daily lives, make assessments before treatment, choose appropriate antihypertensive medications to actively prevent complications (225–229). Moreover, lifestyle modification is important to improve the progression and prognosis of HTN. In addition to genetic and environmental interactions, appropriate lifestyle changes, such as weight loss, healthy diet, reduction of dietary sodium, increased physical activity, and cessation of smoking and excessive alcohol consumption, should be implemented according to the recommendations of the hypertension guidelines (230). However, these non-pharmacological interventions alone are insufficient for HTN treatment. For patients with HTN, salt restriction should be the priority as patients with advanced renal disease who excrete a low sodium load exhibit significant salt and water retention (227, 231). Simultaneously, attention should be paid to the intake of adequate amounts of vegetables and fruits that can protect kidneys from renal injury (232).

## 8 Discussion

In conclusion, inflammation mediates the end-organ damage associated with hypertension. Although reducing blood pressure is crucial, preventing the localized inflammation accompanying this disease, resulting in damage to the vital target organs, should be prioritized (122). In recent years, an increasing number of studies have been published on the pathogenesis of HTN; however, the specific mechanisms are still not fully understood. This review was conducted based on previous studies on the role of immune cells, inflammatory cytokines, and related components in HTN. We analyzed the results at the molecular level to reveal some of the relevant mechanisms to better understand the development of HTN. Future research needs to fill the existing gaps and add more evidence and therapeutic strategies for the prevention and treatment of HTN and avoiding hypertension-related complications.

Hypertension and kidney disease are strongly co-related, and this link should be used effectively to prevent hypertension or treat it at an early stage. Although hypertension is very common today, complications due to hypertension are not encouraging, and many problems remain unresolved. In future studies, antihypertensive drugs, immunosuppressive drugs, and life management must be considered to identify more effective therapeutic targets for HTN.

Although this review focused on inflammatory markers, it is insufficient because the relationship between immunity and inflammation is complex. As the next step, the mechanism of HTN must be explored to alleviate or solve renal injury caused by hypertension.

## Author contributions

XH: Writing – original draft. YL: Writing – review & editing. DH: Conceptualization, Writing – review & editing. YG: Writing – review & editing. XZ: Writing – review & editing. BY: Methodology, Writing – review & editing. JL: Writing – review & editing. XW: Writing – review & editing. KY: Writing – review & editing. JW: Supervision, Writing – review & editing. QL: Resources, Writing – review & editing.
